# Fuzzy Group Consensus Decision Making and Its Use in Selecting Energy-Saving and Low-Carbon Technology Schemes in Star Hotels

**DOI:** 10.3390/ijerph15092057

**Published:** 2018-09-19

**Authors:** Ping Lu, Xuan Yang, Zhou-Jing Wang

**Affiliations:** 1 School of Economic and Management, Xiamen University of Technology, Xiamen 361024, China; luping@xmut.edu.cn; 2 Department of Information Management, Yuan Ze University, Taoyuan 32003, Taiwan; 3 Dongfang College, Zhejiang University of Finance & Economics, Haining 314408, Zhejiang, China; yx_321@zufe.edu.cn; 4 School of Information, Zhejiang University of Finance & Economics, Hangzhou 310018, China

**Keywords:** green tourism, energy-saving and low-carbon, group decision making, consistency index, consensus

## Abstract

Energy-saving and low-carbon technologies play important roles in reducing environmental risk and developing green tourism. An energy-saving and low-carbon technology scheme selection may often involve multiple criteria and sub-criteria as well as multiple stakeholders or decision makers, and thus can be structured as a hierarchical multi-criteria group decision making problem. This paper proposes a framework to solve group consensus decision making problems, where decision makers’ preferences between the alternatives considered with respective to each criterion are elicited by the paired comparison method, and expressed as triangular fuzzy preference relations (TFPRs). The paper first simplifies the existing computation formulas used to determine triangular fuzzy weights of TFPRs. A consistency index is then devised to measure the inconsistency degree of a TFPR and is used to check acceptable consistency of TFPRs. By introducing a possibility degree formula of comparing any two triangular fuzzy weights, an index is defined to measure the consensus level between an individual ranking order and the group ranking order for all alternatives. A consensus model is developed in detail for solving group decision making problems with TFPRs. A case study of selecting energy-saving and low-carbon technology schemes in star hotels is provided to illustrate how to apply the proposed group decision making consensus model in practice.

## 1. Introduction

The tourism industry has become one of the advantageous industries for developing the economy in China. However, this industry usually links a mass of energy consumption and carbon emissions [1,2], and has caused an increasing stress on the environment [3]. To save energy and reduce carbon emissions, the Chinese government has strongly promoted the development of the green economy. Green tourism has been considered an effective solution for energy-saving and environmental protection [4,5]. Constructing an energy-saving and low-carbon system in a star hotel is the fundamental way to respond in green tourism development [6,7]. On the other hand, there often exist different technologies used in constructing energy-saving and low-carbon systems in star hotels. Therefore, it is necessary to select the best one from multiple energy-saving and low-carbon technology schemes when a star hotel wishes to associate with green tourism.

Selection of energy-saving and low-carbon technology schemes in star hotels is frequently based on multiple assessment criteria and involves multiple experts or decision makers. This implies that such a selection can be viewed as a multi-criteria decision making (MCDM) problem with a group of experts. 

The paired comparison method is a popular approach to eliciting decision makers’ preferences or judgments for solving MCDM problems. The judgments in typical paired comparison matrices are characterized by exact ratios. In order to treat with fuzziness of linguistic term-based preferences, Van Laarhoven and Pedrycz [8] proposed the concept of triangular fuzzy numbers and introduced triangular fuzzy preference relations (TFPRs) to model decision makers’ fuzzy judgments. Because decision input information often involves ambiguity, the TFPR based analytic hierarchy process (AHP) (also called fuzzy AHP) has become a common MCDM method, and has triggered a large number of applications in solving real-world decision problems [9].

In group MCDM with paired comparisons, a crucial issue is to check the quality of judgments provided by decision makers, where consistency and acceptable consistency play key roles. For typical paired comparison matrices, Saaty [10] proposed a consistency index (CI) to measure inconsistency degrees and introduced a consistency ratio (CR) to check acceptable consistency. Crawford & Williams [11] put forward another row-geometric-mean-based CI, which was formulated as a geometric consistency index (GCI) in [12]. For TFPRs, different consistency models have been developed in the literature [13,14,15,16]. Recently, Wang [16] used basic triangular fuzzy weights to define consistent TFPRs, and presented computation formulas used to determine triangular fuzzy weights of TFPRs. Some researchers [13,15,16] have pointed out that it is a challenge to develop an appropriate consistency index for measuring inconsistency of TFPRs and checking acceptable consistency of TFPRs. 

Group consensus decision making with paired comparisons involves three different processes. The first process is to check acceptable consistency of individual judgments. The second process called a consensus reaching process is to seek a solution that is sufficiently supported by all decision makers. The last process is to derive a ranking order of all alternatives considered, or to select the best alternative(s). In solving group consensus decision making problems, it is important to develop a consensus model due to the fact that there often exists a large difference among initial judgments provided by decision makers, and thus different ranking orders may be obtained from individual judgments. On the other hand, it is hard to implement a unanimous consensus in solving an actual group decision making problem. A practice method is to use soft consensus measurement [17]. Different soft consensus models have been devised under fuzzy environments [18]. Chiclana et al. [19] gave a comparative study on similarity-based soft consensus models. For hesitant linguistic group decision making, Dong et al. [20] developed a minimum adjustment soft consensus model. Xu et al. [21] proposed a soft consensus model of group decision making with hesitant fuzzy preference relations, and applied it in water allocation management. Soft consensus models of group decision making with intuitionistic fuzzy preference relations can be found in [22,23]. Tan et al. [24] put forward a soft consensus model of group decision making with interval fuzzy preference relations, and used it to solve cooking method selection problems for decreasing organic pollutants in food of animal origin. However, there are two main limitations in the aforementioned soft consensus models: (i) acceptable consistency of individual fuzzy judgments is not enough considered; and (ii) they are based on similarity between individual judgments and aggregated group preferences, implying that the similarity between individual and group decision results is not sufficiently taken into account.

In this paper, we simplify the triangular fuzzy weight computation formulas given in [16]. Based on the simplified expressions, a consistency index is presented to measure the inconsistency degree of a TFPR, and used to check acceptable consistency of TFPRs. We put forward a possibility degree formula for comparing and ranking triangular fuzzy weights. A likelihood degree matrix based index is defined to measure the consensus level between individual and group decision results. Based on the proposed consistency index and consensus index, the paper develops a consensus model for solving group decision making problems with TFPRs.

The remainder of the paper is organized as follows. Section 2 provides the theoretical background, including triangular fuzzy numbers, consistency and triangular fuzzy weights of TFPRs. In Section 3, we simplify the triangular fuzzy weight computation formulas and develop a consistency index for TFPRs. A likelihood degree matrix-based consensus index is defined and a consensus model of group decision making with TFPRs is proposed in Section 4. Section 5 offers a case study of selecting energy-saving and low-carbon technology schemes in star hotels to examine the developed fuzzy group consensus decision making model. Finally, Section 6 draws concluding remarks.

## 2. Theoretical Background

### 2.1. Triangular Fuzzy Numbers and Triangular Fuzzy Preference Relations

The fuzzy set theory introduced by Zadeh [25] uses membership functions to represent possibility distributions of imprecise data. A triangular fuzzy number $\tilde{a}$ is a fuzzy subset of the real line with the following membership function:

(1) $$\mu_{\tilde{a}}(x)=\{\begin{array}{ll} \frac{x-l}{m-l}, & l\leq x\leq m\\ \frac{u-x}{u-m}, & m<x\leq u\\ \begin{array}{l} 0, \end{array} & \begin{array}{l} \mathrm{Otherwise} \end{array} \end{array}\;$$

where m is the modal value of $\tilde{a}$, and l and u are the lower and upper bounds of the support interval of $\tilde{a}$, respectively.

Obviously, $\mu_{\tilde{a}}(x)$ is a triangular and piecewise linear function. Thus, the triangular fuzzy number $\tilde{a}$ can be characterized by a triplet (l,m,u). If l>0, then $\tilde{a}$ is called a positive triangular fuzzy number. In addition, the reciprocal of $\tilde{a}$ is often denoted by ${\tilde{a}}^{c}$, that is, ${\tilde{a}}^{c}=(1/u,\;1/m,\;1/l)$.

Triangular fuzzy numbers are an effective tool for modelling semantic values of linguistic terms. In constructing fuzzy-based decision support systems, we need to assign a set of fuzzy numbers corresponding to a linguistic term set [26,27]. Different triangular fuzzy assignment models (also called triangular fuzzy scales) have been proposed in the literature [28,29,30,31]. Recently, Centobelli et al. [32,33] used trapezoidal fuzzy numbers to assign two linguistic term sets respectively characterizing formalization and sharing degrees of knowledge management tools and knowledge management practices.

In fuzzy MCDM with pairwise comparison matrices, the linguistic term set used must be bipolar, and must have a neutral linguistic term, such as “Indifference” or “Equal importance”. This implies that the set of the codified triangular fuzzy numbers has reciprocity. In other words, for any triangular fuzzy number $\tilde{a}$ in the set, its reciprocal ${\tilde{a}}^{c}$ is also an element of the set.

Once linguistic term-based triangular fuzzy scales have been established, decision makers can use them to elicit paired comparison results, and TFPRs can be employed to describe the decision makers’ fuzzy judgments.

Let $X=\{x_{1},x_{2},...,x_{n}\}$ be a set of considered alternatives. A pairwise comparison matrix $\tilde{A}={\left({\tilde{a}}_{ij}\right)}_{n\times n}={\left(\left(l_{ij},m_{ij},u_{ij}\right)\right)}_{n\times n}$ is said a TFPR on X×X if

(2) $$0<l_{ij}\leq m_{ij}\leq u_{ij},\;l_{ij}u_{ji}=m_{ij}m_{ji}=1,\;l_{ii}=m_{ii}=u_{ii}=1,\;i,j=1,2,...,n\;$$

where ${\tilde{a}}_{ij}=(l_{ij},m_{ij},u_{ij})$ is a positive triangular fuzzy number, and indicates a ratio-based fuzzy preference of alternative $x_{i}$ over $x_{j}$.

Because of efficiency and effectiveness of expressing paired comparison results with vagueness, TFPRs have been widely used in practice, and promoted the theoretical development of fuzzy MCDM [9]. Numerous applications of fuzzy MCDM can be found in the current literature. For instance, Yücenur et al. [34], Lima Junio et al. [35] and Yu et al. [36] used triangular fuzzy MCDM to solve supplier selection problems in green supply chains of industrial industry. Azadeh and Zadeh [37] used a MCDM combined fuzzy analytic hierarchy process to examine maintenance policy selection problems in green manufacturing (also called environmentally conscious manufacturing). Çelikbilek and Tüysüz [38] employed an integrated grey based MCDM method to evaluate renewable energy sources. Grujic et al. [39] applied MCDM in selecting the optimal heat demand in a centralized supply system. Tong and Wang [40] put forward an intuitionistic fuzzy MCDM method for solving low-carbon supplier selection problems. An evaluation indicator system was constructed in Cho et al. [41] by using a fuzzy pairwise comparison-based analytic hierarchy process. Mardani et al. [42] adopted fuzzy MCDM to evaluate energy-saving technologies in five star hotels.

### 2.2. Consistency and Fuzzy Weights of Triangular Fuzzy Preference Relations

For a triangular fuzzy weight vector $\tilde{W}={\left({\tilde{w}}_{1},{\tilde{w}}_{2},...,{\tilde{w}}_{n}\right)}^{T}$ with ${\tilde{w}}_{i}=(w_{i}^{L},w_{i}^{M},w_{i}^{U}),w_{i}^{L}>0$ (i=1,2,...,n), let

(3) $${\tilde{A}}_{\left(\tilde{W}\right)}={\left({\tilde{a}}_{ij}^{\left(\tilde{w}\right)}\right)}_{n\times n}={\left(\left(l_{ij}^{\left(\tilde{w}\right)},m_{ij}^{\left(\tilde{w}\right)},u_{ij}^{\left(\tilde{w}\right)}\right)\right)}_{n\times n},\;$$

where

(4) $$l_{ij}^{\left(\tilde{w}\right)}=\{\begin{array}{ll} 1, & i=j\\ w_{i}^{L}/w_{j}^{U}, & i\neq j \end{array},\;m_{ij}^{\left(\tilde{w}\right)}=\frac{w_{i}^{M}}{w_{j}^{M}},\;u_{ij}^{\left(\tilde{w}\right)}=\{\begin{array}{ll} 1, & i=j\\ w_{i}^{U}/w_{j}^{L}, & i\neq j \end{array}.$$

It is easy to confirm that ${\tilde{A}}_{\left(\tilde{W}\right)}$ satisfies (2) and thus, is a TFPR.

A triangular fuzzy weight vector $\tilde{W}$ is said to be modal-value normalized [16] if the following equation holds true.

(5) $$\prod_{i=1}^{n}w_{i}^{M}=1\;$$

A fuzzy weight vector $\tilde{W}$ is called a basic triangular fuzzy weight vector [16] if the following equation is satisfied.

(6) $$\prod_{i=1}^{n}{\left(w_{i}^{L}w_{i}^{U}\right)}^{1/2}=1\;$$

Wang [16] showed that basic triangular fuzzy weight vectors and modal-value normalized triangular fuzzy weight vectors can be equivalently converted into each other, and thus used the basic triangular fuzzy weight vectors to define consistent TFPRs as follows.

**Definition** **1.** *[16] A TFPR*$\tilde{A}={\left({\tilde{a}}_{ij}\right)}_{n\times n}={\left(\left(l_{ij},m_{ij},u_{ij}\right)\right)}_{n\times n}$*is said to be consistent if there exists a basic triangular fuzzy weight vector such that*${\tilde{A}}_{\left(\tilde{W}\right)}=\tilde{A}$.

In [16], some goal programming models were developed to obtain triangular fuzzy weights denoted by three computation formulas from TFPRs. For any TFPR $\tilde{A}={\left({\tilde{a}}_{ij}\right)}_{n\times n}={\left(\left(l_{ij},m_{ij},u_{ij}\right)\right)}_{n\times n}$ (n≥3), let

(7) $$f_{i}=\prod_{j=1}^{n}\frac{u_{ij}}{l_{ij}},\;i=1,2,...,n\;$$

(8) $$F=\prod_{i=1}^{n}f_{i}=\prod_{i=1}^{n}\prod_{j=1}^{n}\frac{u_{ij}}{l_{ij}}\;$$

(9) $$\phi_{i}^{-}=\min\left\{\frac{{\left(F\right)}^{1/(4(n-1)(n-2))}}{{\left(f_{i}\right)}^{1/(n(n-2))}}{\left(\prod_{j=1}^{n}l_{ij}^{}\right)}^{1/n},\frac{{\left(f_{i}\right)}^{1/(n(n-2))}}{{\left(F\right)}^{1/(4(n-1)(n-2))}}{\left(\prod_{j=1}^{n}u_{ij}^{}\right)}^{1/n}\right\},\;i=1,2,...,n\;$$

(10) $$\phi_{i}^{+}=\max\left\{\frac{{\left(F\right)}^{1/(4(n-1)(n-2))}}{{\left(f_{i}\right)}^{1/(n(n-2))}}{\left(\prod_{j=1}^{n}l_{ij}^{}\right)}^{1/n},\frac{{\left(f_{i}\right)}^{1/(n(n-2))}}{{\left(F\right)}^{1/(4(n-1)(n-2))}}{\left(\prod_{j=1}^{n}u_{ij}^{}\right)}^{1/n}\right\},\;i=1,2,...,n\;$$

(11) $$\rho_{1}=\underset{i}{\max}\left\{\phi_{i}^{-}/{\left(\prod_{j=1}^{n}m_{ij}^{}\right)}^{1/n}\right\},\rho_{2}=\underset{i}{\min}\left\{\phi_{i}^{+}/{\left(\prod_{j=1}^{n}m_{ij}^{}\right)}^{1/n}\right\}\;$$

Then, its triangular fuzzy weight vector ${\tilde{W}}_{H}={\left({\tilde{w}}_{1H},{\tilde{w}}_{2H},...,{\tilde{w}}_{nH}\right)}^{T}$ with ${\tilde{w}}_{iH}=(w_{iH}^{L},w_{iH}^{M},w_{iH}^{U})$ (i=1,2,...,n) is determined as follows.

(12) $$w_{iH}^{L}=\xi_{i}\left(\min\left\{\frac{{\left(F\right)}^{1/(4(n-1)(n-2))}}{{\left(f_{i}\right)}^{1/(n(n-2))}}{\left(\prod_{j=1}^{n}l_{ij}^{}\right)}^{1/n},\frac{{\left(f_{i}\right)}^{1/(n(n-2))}}{{\left(F\right)}^{1/(4(n-1)(n-2))}}{\left(\prod_{j=1}^{n}u_{ij}^{}\right)}^{1/n}\right\}\right)\;$$

(13) $$w_{iH}^{M}=C_{m}{\left(\prod_{j=1}^{n}m_{ij}^{}\right)}^{1/n}\;$$

(14) $$w_{iH}^{U}=\eta_{i}\left(\max\left\{\frac{{\left(F\right)}^{1/(4(n-1)(n-2))}}{{\left(f_{i}\right)}^{1/(n(n-2))}}{\left(\prod_{j=1}^{n}l_{ij}^{}\right)}^{1/n},\frac{{\left(f_{i}\right)}^{1/(n(n-2))}}{{\left(F\right)}^{1/(4(n-1)(n-2))}}{\left(\prod_{j=1}^{n}u_{ij}^{}\right)}^{1/n}\right\}\right)\;$$

where

(15) $$C_{m}=\{\begin{array}{ll} \sqrt{\rho_{1}\rho_{2}}, & \left(\rho_{1}\leq \rho_{2}<1)\;\vee \;(1<\rho_{1}\leq \rho_{2}\right)\\ 1, & \mathrm{Otherwise}\; \end{array}\;$$

(16) $$\xi_{i}=\{\begin{array}{ll} {\left(\prod_{j=1}^{n}m_{ij}^{}\right)}^{1/n}/\phi_{i}^{-}, & (\rho_{2}<\rho_{1})\wedge \left({\left(\prod_{j=1}^{n}m_{ij}^{}\right)}^{1/n}<\phi_{i}^{-}\right)\\ 1, & \mathrm{Otherwise}\; \end{array}\;$$

(17) $$\eta_{i}=\{\begin{array}{ll} {\left(\prod_{j=1}^{n}m_{ij}^{}\right)}^{1/n}/\phi_{i}^{+}, & (\rho_{2}<\rho_{1})\wedge \left({\left(\prod_{j=1}^{n}m_{ij}^{}\right)}^{1/n}>\phi_{i}^{+}\right)\\ 1, & \mathrm{Otherwise}\; \end{array}\;$$

It has been shown in [16] that if $f_{i}\geq {\left(F\right)}^{1/(2n-2)},\forall i=1,2,...,n.$, then ${\tilde{w}}_{iH}=(w_{iH}^{L},w_{iH}^{M},w_{iH}^{U})$ (i=1,2,...,n) defined by (12)–(14) are optimized triangular fuzzy weights derived from $\tilde{A}$. Moreover, the following important result can be used to judge whether a TFPR is consistent under Definition 1.

**Lemma** **1.** [16] *A* *TFPR* $\tilde{A}={\left({\tilde{a}}_{ij}\right)}_{n\times n}={\left(\left(l_{ij},m_{ij},u_{ij}\right)\right)}_{n\times n}$ *(*n≥3*) is consistent if and only if* $\tilde{A}={\tilde{A}}_{\left({\tilde{W}}_{H}\right)}$*, where* ${\tilde{A}}_{\left({\tilde{W}}_{H}\right)}$ *is defined by (3) and (4).*

## 3. Inconsistency Measurement for Triangular Fuzzy Preference Relations

This section first simplifies the three computation Formulas (12)–(14). A consistency index of TFPRs is then introduced to measure the inconsistency degree of a TFPR.

Let

(18) $$\Phi_{i}=\frac{\max\left\{{\left(f_{i}\right)}^{1/(n-2)},{\left(F\right)}^{1/(2(n-1)(n-2))}\right\}}{\min\left\{{\left(f_{i}\right)}^{1/(n-2)},{\left(F\right)}^{1/(2(n-1)(n-2))}\right\}},\;i=1,2,...,n$$

Obviously, $\Phi_{i}\geq 1$ for all i=1,2,...,n. Then we have following result.

**Theorem** **1.** *Let* $\tilde{A}={\left({\tilde{a}}_{ij}\right)}_{n\times n}={\left(\left(l_{ij},m_{ij},u_{ij}\right)\right)}_{n\times n}$ *(* n≥3 *) be a TFPR, then*

(19) $$\phi_{i}^{-}/{\left(\prod_{j=1}^{n}m_{ij}^{}\right)}^{1/n}={\left(\prod_{j=1}^{n}\frac{l_{ij}^{}u_{ij}^{}}{{\left(m_{ij}^{}\right)}^{2}}\right)}^{1/(2n)}/{\left(\Phi_{i}\right)}^{1/2},\;i=1,2,...,n\;$$

(20) $$\phi_{i}^{+}/{\left(\prod_{j=1}^{n}m_{ij}^{}\right)}^{1/n}={\left(\Phi_{i}\right)}^{1/2}{\left(\prod_{j=1}^{n}\frac{l_{ij}^{}u_{ij}^{}}{{\left(m_{ij}^{}\right)}^{2}}\right)}^{1/(2n)},\;i=1,2,...,n$$

**Proof.**  If $f_{i}\geq {\left(F\right)}^{1/(2n-2)}$, it follows from (18) that $\Phi_{i}=\frac{{\left(f_{i}\right)}^{1/(n-2)}}{{\left(F\right)}^{1/(2(n-1)(n-2))}}$. As per (9) and (10), one can obtain $\phi_{i}^{-}=\frac{{\left(F\right)}^{1/(4(n-1)(n-2))}}{{\left(f_{i}\right)}^{1/(n(n-2))}}{\left(\prod_{j=1}^{n}l_{ij}^{}\right)}^{1/n}$ and $\phi_{i}^{+}=\frac{{\left(f_{i}\right)}^{1/(n(n-2))}}{{\left(F\right)}^{1/(4(n-1)(n-2))}}{\left(\prod_{j=1}^{n}u_{ij}^{}\right)}^{1/n}$. Thus,

$$\begin{array}{l} \phi_{i}^{-}/{\left(\prod_{j=1}^{n}m_{ij}^{}\right)}^{1/n}=\frac{{\left(F\right)}^{1/(4(n-1)(n-2))}}{{\left(f_{i}\right)}^{1/(n(n-2))}}{\left(\prod_{j=1}^{n}\frac{l_{ij}^{}}{m_{ij}^{}}\right)}^{1/n}=\frac{{\left(f_{i}\right)}^{1/(2n)}}{{\left(\Phi_{i}\right)}^{1/2}}{\left(\prod_{j=1}^{n}\frac{l_{ij}^{}}{m_{ij}^{}}\right)}^{1/n}\\ =\frac{{\left(\prod_{j=1}^{n}\frac{u_{ij}^{}}{l_{ij}^{}}\right)}^{1/(2n)}}{{\left(\Phi_{i}\right)}^{1/2}}{\left(\prod_{j=1}^{n}\frac{l_{ij}^{}}{m_{ij}^{}}\right)}^{1/n}={\left(\prod_{j=1}^{n}\frac{l_{ij}^{}u_{ij}^{}}{{\left(m_{ij}^{}\right)}^{2}}\right)}^{1/(2n)}/{\left(\Phi_{i}\right)}^{1/2}, \end{array}$$

$$\begin{array}{l} \phi_{i}^{+}/{\left(\prod_{j=1}^{n}m_{ij}^{}\right)}^{1/n}=\frac{{\left(f_{i}\right)}^{1/(n(n-2))}}{{\left(F\right)}^{1/(4(n-1)(n-2))}}{\left(\prod_{j=1}^{n}\frac{u_{ij}^{}}{m_{ij}^{}}\right)}^{1/n}=\frac{{\left(\Phi_{i}\right)}^{1/2}}{{\left(f_{i}\right)}^{1/(2n)}}{\left(\prod_{j=1}^{n}\frac{u_{ij}^{}}{m_{ij}^{}}\right)}^{1/n}\\ =\frac{{\left(\Phi_{i}\right)}^{1/2}}{{\left(\prod_{j=1}^{n}\frac{u_{ij}^{}}{l_{ij}^{}}\right)}^{1/(2n)}}{\left(\prod_{j=1}^{n}\frac{u_{ij}^{}}{m_{ij}^{}}\right)}^{1/n}={\left(\Phi_{i}\right)}^{1/2}{\left(\prod_{j=1}^{n}\frac{l_{ij}^{}u_{ij}^{}}{{\left(m_{ij}^{}\right)}^{2}}\right)}^{1/(2n)}. \end{array}$$

Similarly, (19) and (20) hold true because $\Phi_{i}=\frac{{\left(F\right)}^{1/(2(n-1)(n-2))}}{{\left(f_{i}\right)}^{1/(n-2)}}$ if $f_{i}<{\left(F\right)}^{1/(2n-2)}$. Thus, the proof of Theorem 1 is completed. □

Based on Theorem 1, (11) can be equivalently expressed as

(21) $$\rho_{1}=\underset{i}{\max}\left\{{\left(\prod_{j=1}^{n}\frac{l_{ij}^{}u_{ij}^{}}{{\left(m_{ij}^{}\right)}^{2}}\right)}^{1/(2n)}/{\left(\Phi_{i}\right)}^{1/2}\right\},\;\rho_{2}=\underset{i}{\min}\left\{{\left(\Phi_{i}\right)}^{1/2}{\left(\prod_{j=1}^{n}\frac{l_{ij}^{}u_{ij}^{}}{{\left(m_{ij}^{}\right)}^{2}}\right)}^{1/(2n)}\right\}.\;$$

Let

(22) $$\Theta_{i}=\{\begin{array}{ll} {\left(\prod_{j=1}^{n}\frac{l_{ij}^{}u_{ij}^{}}{{\left(m_{ij}^{}\right)}^{2}}\right)}^{1/n}, & \left(\rho_{1}>\rho_{2}\right)\wedge \left({\left(\prod_{j=1}^{n}\frac{l_{ij}^{}u_{ij}^{}}{{\left(m_{ij}^{}\right)}^{2}}\right)}^{1/n}>\Phi_{i}\right)\\ \Phi_{i}, & \mathrm{Otherwise}\; \end{array},\;i=1,2,...,n\;$$

(23) $$\theta_{i}=\{\begin{array}{ll} {\left(\prod_{j=1}^{n}\frac{{\left(m_{ij}^{}\right)}^{2}}{l_{ij}^{}u_{ij}^{}}\right)}^{1/n}, & \left(\rho_{1}>\rho_{2}\right)\wedge \left({\left(\prod_{j=1}^{n}\frac{{\left(m_{ij}^{}\right)}^{2}}{l_{ij}^{}u_{ij}^{}}\right)}^{1/n}>\Phi_{i}\right)\\ \Phi_{i}, & \mathrm{Otherwise}\; \end{array},\;i=1,2,...,n\;$$

Then, we have the following theorem.

**Theorem** **2.** *Let*$\tilde{A}={\left({\tilde{a}}_{ij}\right)}_{n\times n}={\left(\left(l_{ij},m_{ij},u_{ij}\right)\right)}_{n\times n}$*(*n≥3*) be a TFPR, then*${\tilde{w}}_{iH}=(w_{iH}^{L},w_{iH}^{M},w_{iH}^{U})$*(*i=1,2,...,n) *defined by (12)–(14) can be equivalently expressed as
*

(24) $$w_{iH}^{L}=\frac{{\left(f_{i}\right)}^{1/(2n)}}{{\left(\Theta_{i}\right)}^{1/2}}{\left(\prod_{j=1}^{n}l_{ij}\right)}^{1/n},\;w_{iH}^{M}=C_{m}{\left(\prod_{j=1}^{n}m_{ij}\right)}^{1/n},\;w_{iH}^{U}=\frac{{\left(\theta_{i}\right)}^{1/2}}{{\left(f_{i}\right)}^{1/(2n)}}{\left(\prod_{j=1}^{n}u_{ij}\right)}^{1/n}\;$$

*where*$f_{i}$*,*$C_{m}$*,*$\Theta_{i}$*and*$\theta_{i}$*are defined by (7), (15), (22) and (23), respectively.*

**Proof.**  It is obvious that the second formula in (24) is the same as (13). Next, we prove that (12) and (14) can be respectively rewritten as the first formula in (24) and the last formula in (24) by considering the following three cases.

Case 1: $(\rho_{1}\leq \rho_{2})\vee \left((\rho_{1}>\rho_{2})\wedge \left(\frac{1}{\Phi_{i}}\leq {\left(\prod_{j=1}^{n}\frac{l_{ij}^{}u_{ij}^{}}{{\left(m_{ij}^{}\right)}^{2}}\right)}^{1/n}\leq \Phi_{i}\right)\right)$. In this case, $\Theta_{i}=\theta_{i}=\Phi_{i}$. As per (16) and (17), we have $\xi_{i}=\eta_{i}=1$. It follows from (9), (10), (12) and (14) that

(25) $$w_{iH}^{L}=\xi_{i}\phi_{i}^{-}=\phi_{i}^{-}={\left(\prod_{j=1}^{n}m_{ij}^{}\right)}^{1/n}{\left(\prod_{j=1}^{n}\frac{l_{ij}^{}u_{ij}^{}}{{\left(m_{ij}^{}\right)}^{2}}\right)}^{1/(2n)}/{\left(\Phi_{i}\right)}^{1/2}=\frac{{\left(f_{i}\right)}^{1/(2n)}}{{\left(\Theta_{i}\right)}^{1/2}}{\left(\prod_{j=1}^{n}l_{ij}\right)}^{1/n}\;$$

(26) $$w_{iH}^{U}=\eta_{i}\phi_{i}^{+}=\phi_{i}^{+}={\left(\prod_{j=1}^{n}m_{ij}^{}\right)}^{1/n}{\left(\Phi_{i}\right)}^{1/2}{\left(\prod_{j=1}^{n}\frac{l_{ij}^{}u_{ij}^{}}{{\left(m_{ij}^{}\right)}^{2}}\right)}^{1/(2n)}=\frac{{\left(\theta_{i}\right)}^{1/2}}{{\left(f_{i}\right)}^{1/(2n)}}{\left(\prod_{j=1}^{n}u_{ij}\right)}^{1/n}\;$$

where the third equality in (25) is derived from (19), and the third equality in (26) is obtained from (20) in Theorem 1.

Case 2: $(\rho_{1}>\rho_{2})\wedge \left({\left(\prod_{j=1}^{n}\frac{l_{ij}^{}u_{ij}^{}}{{\left(m_{ij}^{}\right)}^{2}}\right)}^{1/n}>\Phi_{i}\right)$. In this case, it directly follows from (22), (23) and $\Phi_{i}\geq 1$ that $\Theta_{i}={\left(\prod_{j=1}^{n}\frac{l_{ij}^{}u_{ij}^{}}{{\left(m_{ij}^{}\right)}^{2}}\right)}^{1/n}$ and $\theta_{i}=\Phi_{i}$. Thus, we have $\Theta_{i}>\Phi_{i}$. 

According to Theorem 1, one gets

$$\begin{array}{l} \phi_{i}^{-}/{\left(\prod_{j=1}^{n}m_{ij}^{}\right)}^{1/n}=\left({\left(\prod_{j=1}^{n}\frac{l_{ij}^{}u_{ij}^{}}{{\left(m_{ij}^{}\right)}^{2}}\right)}^{1/(2n)}/{\left(\Phi_{i}\right)}^{1/2}\right)>\left({\left(\prod_{j=1}^{n}\frac{l_{ij}^{}u_{ij}^{}}{{\left(m_{ij}^{}\right)}^{2}}\right)}^{1/(2n)}/{\left(\Theta_{i}\right)}^{1/2}\right)=1\\ \Rightarrow \phi_{i}^{-}>{\left(\prod_{j=1}^{n}m_{ij}^{}\right)}^{1/n}, \end{array}$$

$$\phi_{i}^{+}/{\left(\prod_{j=1}^{n}m_{ij}^{}\right)}^{1/n}\geq \phi_{i}^{-}/{\left(\prod_{j=1}^{n}m_{ij}^{}\right)}^{1/n}>1\Rightarrow \phi_{i}^{+}>{\left(\prod_{j=1}^{n}m_{ij}^{}\right)}^{1/n}.\;$$

As per (16) and (17), we obtain $\xi_{i}={\left(\prod_{j=1}^{n}m_{ij}^{}\right)}^{1/n}/\phi_{i}^{-}$ and $\eta_{i}=1$. By (9), (10), (12) and (14), one has

(27) $$w_{iH}^{L}=\xi_{i}\phi_{i}^{-}={\left(\prod_{j=1}^{n}m_{ij}^{}\right)}^{1/n}=\frac{{\left(f_{i}\right)}^{1/(2n)}}{{\left(\prod_{j=1}^{n}\frac{l_{ij}^{}u_{ij}^{}}{{\left(m_{ij}^{}\right)}^{2}}\right)}^{1/(2n)}}{\left(\prod_{j=1}^{n}l_{ij}\right)}^{1/n}=\frac{{\left(f_{i}\right)}^{1/(2n)}}{{\left(\Theta_{i}\right)}^{1/2}}{\left(\prod_{j=1}^{n}l_{ij}\right)}^{1/n},\;$$

(28) $$w_{iH}^{U}=\eta_{i}\phi_{i}^{+}=\phi_{i}^{+}={\left(\prod_{j=1}^{n}m_{ij}^{}\right)}^{1/n}{\left(\Phi_{i}\right)}^{1/2}{\left(\prod_{j=1}^{n}\frac{l_{ij}^{}u_{ij}^{}}{{\left(m_{ij}^{}\right)}^{2}}\right)}^{1/(2n)}=\frac{{\left(\theta_{i}\right)}^{1/2}}{{\left(f_{i}\right)}^{1/(2n)}}{\left(\prod_{j=1}^{n}u_{ij}\right)}^{1/n},\;$$

where the last equality in (28) is confirmed by $\theta_{i}=\Phi_{i}$, and the third equality in (28) is obtained from (20).

Case 3: $\left(\rho_{1}>\rho_{2}\right)\wedge \left({\left(\prod_{j=1}^{n}\frac{{\left(m_{ij}^{}\right)}^{2}}{l_{ij}^{}u_{ij}^{}}\right)}^{1/n}>\Phi_{i}\right)$. In this case, as per (22), (23) and $\Phi_{i}\geq 1$, we have $\Theta_{i}=\Phi_{i}$ and $\theta_{i}={\left(\prod_{j=1}^{n}\frac{{\left(m_{ij}^{}\right)}^{2}}{l_{ij}^{}u_{ij}^{}}\right)}^{1/n}$. Therefore, one has $\theta_{i}>\Phi_{i}$

According to (19) and (20), we obtain

$$\phi_{i}^{+}/{\left(\prod_{j=1}^{n}m_{ij}^{}\right)}^{1/n}={\left(\Phi_{i}\right)}^{1/2}{\left(\prod_{j=1}^{n}\frac{l_{ij}^{}u_{ij}^{}}{{\left(m_{ij}^{}\right)}^{2}}\right)}^{1/(2n)}<{\left(\theta_{i}\right)}^{1/2}{\left(\prod_{j=1}^{n}\frac{l_{ij}^{}u_{ij}^{}}{{\left(m_{ij}^{}\right)}^{2}}\right)}^{1/(2n)}=1\Rightarrow \phi_{i}^{+}<{\left(\prod_{j=1}^{n}m_{ij}^{}\right)}^{1/n},$$

$$\phi_{i}^{-}/{\left(\prod_{j=1}^{n}m_{ij}^{}\right)}^{1/n}\leq \phi_{i}^{+}/{\left(\prod_{j=1}^{n}m_{ij}^{}\right)}^{1/n}<1.$$

According to (16) and (17), one gets $\xi_{i}=1$ and $\eta_{i}={\left(\prod_{j=1}^{n}m_{ij}^{}\right)}^{1/n}/\phi_{i}^{+}$. It follows from (9), (10), (12) and (14) that

(29) $$w_{iH}^{L}=\xi_{i}\phi_{i}^{-}=\phi_{i}^{-}={\left(\prod_{j=1}^{n}m_{ij}^{}\right)}^{1/n}{\left(\prod_{j=1}^{n}\frac{l_{ij}^{}u_{ij}^{}}{{\left(m_{ij}^{}\right)}^{2}}\right)}^{1/(2n)}/{\left(\Phi_{i}\right)}^{1/2}=\frac{{\left(f_{i}\right)}^{1/(2n)}}{{\left(\Theta_{i}\right)}^{1/2}}{\left(\prod_{j=1}^{n}l_{ij}\right)}^{1/n},\;$$

(30) $$w_{iH}^{U}=\eta_{i}\phi_{i}^{+}={\left(\prod_{j=1}^{n}m_{ij}^{}\right)}^{1/n}=\left({\left(\prod_{j=1}^{n}\frac{{\left(m_{ij}^{}\right)}^{2}}{l_{ij}^{}u_{ij}^{}}\right)}^{1/(2n)}{\left(\prod_{j=1}^{n}u_{ij}\right)}^{1/n}\right)/{\left(\prod_{j=1}^{n}\frac{u_{ij}^{}}{l_{ij}^{}}\right)}^{1/(2n)}=\frac{{\left(\theta_{i}\right)}^{1/2}}{{\left(f_{i}\right)}^{1/(2n)}}{\left(\prod_{j=1}^{n}u_{ij}\right)}^{1/n},\;$$

where the last equality in (29) is obtained from $\Theta_{i}=\Phi_{i}$, and the third equality in (29) is derived from (19) in Theorem 1.

Therefore, we complete the proof of Theorem 2. □

According to (5), it is easy to confirm that if $C_{m}=1$, then the triangular fuzzy weight vector ${\tilde{W}}_{H}={\left({\tilde{w}}_{1H},{\tilde{w}}_{2H},...,{\tilde{w}}_{nH}\right)}^{T}$ with ${\tilde{w}}_{iH}=(w_{iH}^{L},w_{iH}^{M},w_{iH}^{U})$ (i=1,2,...,n) defined by (24) is modal-value normalized. If $\Theta_{i}=\theta_{i}$ for all i=1,2,...,n, then ${\tilde{W}}_{H}$ is a basic triangular fuzzy weight vector. If $C_{m}=1$ and $\Theta_{i}=\theta_{i},\forall i=1,2,...,n$, then ${\tilde{W}}_{H}$ is a normalized basic triangular fuzzy weight vector.

Motivated by the geometric inconsistency measurement model for pairwise comparison matrices presented by Crawford and Williams [11], we define the following consistency index to measure the inconsistency degree of a TFPR.

**Definition** **2.** *Given a TFPR* $\tilde{A}={\left({\tilde{a}}_{ij}\right)}_{n\times n}={\left(\left(l_{ij},m_{ij},u_{ij}\right)\right)}_{n\times n}$ *, and* *the* *triangular fuzzy weight* *s* ${\tilde{w}}_{iH}=(w_{iH}^{L},w_{iH}^{M},w_{iH}^{U})$ *(* i=1,2,...,n *) obtained by (24), a consistency index (CI) is defined as*

(31) $$\overset{︹}{CI}(\tilde{A})=\frac{2}{3(n-1)(n-2)}\sum_{i=1}^{n-1}\sum_{j=i+1}^{n}\left(\begin{array}{l} {\left(\ln l_{ij}-\ln(w_{iH}^{L}/w_{jH}^{U})\right)}^{2}+{\left(\ln m_{ij}-\ln(w_{iH}^{M}/w_{jH}^{M})\right)}^{2}\\ +{\left(\ln u_{ij}-\ln(w_{iH}^{U}/w_{jH}^{L})\right)}^{2} \end{array}\right)\;$$

Obviously, $\overset{︹}{CI}(\tilde{A})\geq 0$. The first part ${\left(\ln l_{ij}-\ln(w_{iH}^{L}/w_{jH}^{U})\right)}^{2}$ is the squared distance between the log of the lower bound of the support interval of the fuzzy judgment ${\tilde{a}}_{ij}$ and the log of the value $w_{iH}^{L}/w_{jH}^{U}$. The second part in (31) is squared distance between the log of the modal value of the fuzzy judgment ${\tilde{a}}_{ij}$ and the log of the ratio $w_{iH}^{M}/w_{jH}^{M}$. The last part is the squared distance between the log of the upper bound of the support interval of the fuzzy judgment ${\tilde{a}}_{ij}$ and the log of the value $w_{iH}^{L}/w_{jH}^{U}$. This implies that $\overset{︹}{CI}(\tilde{A})$ gives a distance between the two TFPRs $\tilde{A}$ and ${\tilde{A}}_{\left({\tilde{W}}_{H}\right)}$, where ${\tilde{W}}_{H}={\left({\tilde{w}}_{1H},{\tilde{w}}_{2H},...,{\tilde{w}}_{nH}\right)}^{T}$ and ${\tilde{A}}_{\left({\tilde{W}}_{H}\right)}$ is defined by (3) and (4). As per Lemma 1, $\overset{︹}{CI}(\tilde{A})=0$ if $\tilde{A}$ is consistent. The bigger the value $\overset{︹}{CI}(\tilde{A})$, the stronger the inconsistency level of the fuzzy judgments in $\tilde{A}$.

According to (24) and the reciprocity of $l_{ij}u_{ji}=1,\forall i,j=1,2,...,n$, $\overset{︹}{CI}(\tilde{A})$ can be equivalently expressed as

(32) $$\overset{︹}{CI}(\tilde{A})=\frac{2}{3(n-1)(n-2)}\sum_{i=1}^{n-1}\sum_{j=i+1}^{n}\left(\begin{array}{l} {\left(\ln l_{ij}-\frac{1}{n}\sum_{k=1}^{n}\ln(l_{ik}l_{kj})+\frac{1}{2}(\ln\Theta_{i}-\ln\theta_{j})-\frac{1}{2n}(\ln f_{i}-\ln f_{j})\right)}^{2}\\ +{\left(\ln m_{ij}-\frac{1}{n}\sum_{k=1}^{n}\ln(m_{ik}m_{kj})\right)}^{2}\\ +{\left(\log u_{ij}-\frac{1}{n}\sum_{k=1}^{n}\ln(u_{ik}u_{kj})+\frac{1}{2}(\ln\Theta_{j}-\ln\theta_{i})-\frac{1}{2n}(\ln f_{j}-\ln f_{i})\right)}^{2} \end{array}\right)\;$$

**Definition** **3.** *Given a TFPR*$\tilde{A}={\left({\tilde{a}}_{ij}\right)}_{n\times n}={\left(\left(l_{ij},m_{ij},u_{ij}\right)\right)}_{n\times n}$*, and an acceptable consistency threshold*$t_{\delta }$*(*$t_{\delta }>0$*), then*$\tilde{A}$*is acceptable if*$\overset{︹}{CI}(\tilde{A})\leq t_{\delta }$.

If a FFPR $\tilde{A}={\left({\tilde{a}}_{ij}\right)}_{n\times n}={\left(\left(l_{ij},m_{ij},u_{ij}\right)\right)}_{n\times n}$ becomes an original comparison matrix [10], that is, $l_{ij}=m_{ij}=u_{ij}$ for all i,j=1,2,...,n, then by (7), (22) and (23), we have $f_{i}=1$, $\Theta_{i}=1$ and $\theta_{i}=1$ for each i=1,2,...,n. In this case, $\overset{︹}{CI}(\tilde{A})=\frac{2}{\left(n-1)(n-2\right)}\sum_{i=1}^{n-1}\sum_{j=i+1}^{n}{\left(\ln m_{ij}-\frac{1}{n}\sum_{k=1}^{n}\ln(m_{ik}m_{kj})\right)}^{2}$, which is the same as the geometric inconsistency index reformulated by Aguaron and Moreno-Jimenez [12]. This shows that the approximated thresholds given in [12] can be used to check acceptable consistency of TFPRs. These thresholds are shown in Table 1.

## 4. A Group Decision Making Consensus Model Based on Triangular Fuzzy Preference Relations

This section introduces a possibility degree formula to compare any two positive triangular fuzzy weights. An index is defined to measure the consensus levels of individual TFPRs, and a consensus model is developed to solve group MCDM problems with TFPRs.

For a group decision making problem with a set of decision makers $D=\left\{d_{1},d_{2},...,d_{m}\right\}$, each decision maker $d_{p}$ (p=1,2,...,m) carries out pairwise comparisons on X, and provides a TFPR ${\tilde{A}}_{\left(p\right)}={\left({\tilde{a}}_{ij(p)}\right)}_{n\times n}={\left(\left(l_{ij(p)},m_{ij(p)},u_{ij(p)}\right)\right)}_{n\times n}$ to describe his/her fuzzy judgments. As per (3.7), one can obtain a triangular fuzzy weight vector denoted by ${\tilde{W}}_{H}^{\left(p\right)}={\left({\tilde{w}}_{1H}^{\left(p\right)},{\tilde{w}}_{2H}^{\left(p\right)},...,{\tilde{w}}_{nH}^{\left(p\right)}\right)}^{T}$ with ${\tilde{w}}_{iH}^{\left(p\right)}=(w_{iH}^{L(p)},w_{iH}^{M(p)},w_{iH}^{U(p)})$ (i=1,2,...,n) for each of ${\tilde{A}}_{\left(p\right)}$ (p=1,2,...,m).

Assume that the importance weight of the decision maker $d_{p}$ is $\lambda_{p}$, where $0<\lambda_{p}<1$ and $\sum_{p=1}^{n}\lambda_{p}=1$, then a group TFPR ${\tilde{A}}_{\left(G\right)}={\left({\tilde{a}}_{ij(G)}\right)}_{n\times n}={\left(\left(l_{ij(G)},m_{ij(G)},u_{ij(G)}\right)\right)}_{n\times n}$ is obtained by using the following aggregation method.

(33) $$l_{ij(G)}=\prod_{p=1}^{m}{\left(l_{ij(p)}\right)}^{\lambda_{p}},\;m_{ij(G)}=\prod_{p=1}^{m}{\left(m_{ij(p)}\right)}^{\lambda_{p}},\;u_{ij(G)}=\prod_{p=1}^{m}{\left(u_{ij(p)}\right)}^{\lambda_{p}}\;$$

According to (24), a group triangular fuzzy weight vector is derived and denoted by ${\tilde{W}}_{H}^{\left(G\right)}={\left({\tilde{w}}_{1H}^{\left(G\right)},{\tilde{w}}_{2H}^{\left(G\right)},...,{\tilde{w}}_{nH}^{\left(G\right)}\right)}^{T}$ with ${\tilde{w}}_{iH}^{\left(G\right)}=(w_{iH}^{L(G)},w_{iH}^{M(G)},w_{iH}^{U(G)})$ (i=1,2,...,n).

In order to compare and rank triangular fuzzy weights obtained from TFPRs by using the computation Formula (24), a possibility degree formula is introduced as follows.

**Definition** **4.** *Given any two* *triangular fuzzy weight* *s* ${\tilde{w}}_{\alpha }=(w_{\alpha }^{L},w_{\alpha }^{M},w_{\alpha }^{U})$ *and* ${\tilde{w}}_{\beta }=(w_{\beta }^{L},w_{\beta }^{M},w_{\beta }^{U})$ *, the likelihood degree of* ${\tilde{w}}_{\alpha }$ *being* *no less than* ${\tilde{w}}_{\beta }$ *is defined as*

(34) $$L({\tilde{w}}_{\alpha }^{}\geq {\tilde{w}}_{\beta }^{})=\frac{\max\left\{0,\ln w_{\alpha }^{M}+\ln w_{\alpha }^{U}-\ln w_{\beta }^{L}-\ln w_{\beta }^{M}\right\}-\min\left\{0,\ln w_{\alpha }^{L}+\ln w_{\alpha }^{M}-\ln w_{\beta }^{M}-\ln w_{\beta }^{U}\right\}}{\ln w_{\alpha }^{U}-\ln w_{\alpha }^{L}+\ln w_{\beta }^{U}-\ln w_{\beta }^{L}}\;$$

As $0<w_{\alpha }^{L}\leq w_{\alpha }^{M}\leq w_{\alpha }^{U}$ and $0<w_{\beta }^{L}\leq w_{\beta }^{M}\leq w_{\beta }^{U}$, one has $0\leq P({\tilde{w}}_{\alpha }^{}\geq {\tilde{w}}_{\beta }^{})\leq 1$, $P({\tilde{w}}_{\alpha }^{}\geq {\tilde{w}}_{\alpha }^{})=1$ and $P({\tilde{w}}_{\alpha }^{}\geq {\tilde{w}}_{\beta }^{})+P({\tilde{w}}_{\beta }^{}\geq {\tilde{w}}_{\alpha }^{})=1$.

Based on (34) and the obtained triangular fuzzy weights ${\tilde{w}}_{iH}^{\left(p\right)}=(w_{iH}^{L(p)},w_{iH}^{M(p)},w_{iH}^{U(p)})$ (i=1,2,...,n), a likelihood degree matrix can be established as

(35) $$L_{p}={\left(z_{ij}^{\left(p\right)}\right)}_{n\times n}={\left(L({\tilde{w}}_{iH}^{\left(p\right)}\geq {\tilde{w}}_{jH}^{\left(p\right)})\right)}_{n\times n}\;$$

for each p=1,2,...,m.

Similarly, based on the group triangular fuzzy weights ${\tilde{w}}_{iH}^{\left(G\right)}=(w_{iH}^{L(G)},w_{iH}^{M(G)},w_{iH}^{U(G)})$ (i=1,2,...,n), a group likelihood degree matrix is established as

(36) $$L_{G}={\left(z_{ij}^{\left(G\right)}\right)}_{n\times n}={\left(L({\tilde{w}}_{iH}^{\left(G\right)}\geq {\tilde{w}}_{jH}^{\left(G\right)})\right)}_{n\times n}\;$$

Based on (35) and (36), an index is introduced to measure the consensus of individual judgments with respect to and the group result.

**Definition** **5.** *Let* $L_{p}={\left(z_{ij}^{\left(p\right)}\right)}_{n\times n}$ *(* p=1,2,...,m *) be the likelihood degree matrix defined by (35), and* $L_{G}={\left(z_{ij}^{\left(G\right)}\right)}_{n\times n}$ *be the group likelihood degree matrix defined by (36), the consensus index of the individual judgments in* ${\tilde{A}}_{\left(p\right)}$ *with respective to the group result is defined as*

(37) $$CD(L_{p})=1-\frac{2}{n(n-1)}\sum_{i=1}^{n-1}\sum_{j=i+1}^{n}\left|z_{ij}^{\left(p\right)}-z_{ij}^{\left(G\right)}\right|\;$$

Obviously, $0\leq CD(L_{p})\leq 1$ for each p=1,2,...,m. If $CD(L_{p})=1$, then $L_{p}=L_{G}$, implying that the likelihood of the ranking order of any two alternatives based on ${\tilde{A}}_{\left(p\right)}$ is the same as that based on ${\tilde{A}}_{\left(G\right)}$, and the ranking orders of all alternatives obtained from ${\tilde{A}}_{\left(p\right)}$ and ${\tilde{A}}_{\left(G\right)}$ are fully identical. The bigger the value of $CD(L_{p})$, the stronger the consensus between the individual ranking order with likelihoods and the group result.

Let $c_{\delta }$($0<c_{\delta }\leq 1$) be an acceptable consensus threshold, if $CD(L_{p})\geq c_{\delta }$ for all p=1,2,...,m, then there is a consensus result among the *m* decision makers. If $CD(L_{p})<c_{\delta }$(p∈{1,2,...,m}), then there does not exist a consensus among the *m* decision makers. In this case, the fuzzy judgments in ${\tilde{A}}_{\left(p\right)}$ should be returned to the decision maker $d_{p}$ for a re-statement.

Based on the aforesaid analysis, we devise an acceptable consistency and acceptable consensus-based group decision making procedure as follows.

Step 1: Employ the computation Formula (32) to compute the inconsistency index $\overset{︹}{CI}({\tilde{A}}_{\left(p\right)})$ for each of the individual TFPRs ${\tilde{A}}_{\left(p\right)}$(p=1,2,...,m).

Step 2: Check acceptable consistency of ${\tilde{A}}_{\left(p\right)}$(p=1,2,...,m) according to Definition 3. If all individual TFPRs ${\tilde{A}}_{\left(p\right)}$(p=1,2,...,m) are of acceptable consistency, then go to the next step; otherwise, return the unacceptable TFPR ${\tilde{A}}_{\left(q\right)}$ (q∈{1,2,...,m}) to the decision maker $d_{q}$ for a revision and go to step 10.

Step 3: Aggregate individual TFPRs ${\tilde{A}}_{\left(p\right)}={\left({\tilde{a}}_{ij(p)}\right)}_{n\times n}={\left(\left(l_{ij(p)},m_{ij(p)},u_{ij(p)}\right)\right)}_{n\times n}$ (p=1,2,...,m) into a group TFPR ${\tilde{A}}_{\left(G\right)}={\left({\tilde{a}}_{ij(G)}\right)}_{n\times n}={\left(\left(l_{ij(G)},m_{ij(G)},u_{ij(G)}\right)\right)}_{n\times n}$ as per (33).

Step 4: Utilize the computation Formula (24) to obtain triangular fuzzy weights ${\tilde{w}}_{iH}^{\left(p\right)}=(w_{iH}^{L(p)},w_{iH}^{M(p)},w_{iH}^{U(p)})$ (i=1,2,...,n) from each of the individual TFPRs ${\tilde{A}}_{\left(p\right)}$(p=1,2,...,m), and to derive the group fuzzy weights ${\tilde{w}}_{iH}^{\left(G\right)}=(w_{iH}^{L(G)},w_{iH}^{M(G)},w_{iH}^{U(G)})$ (i=1,2,...,n) from ${\tilde{A}}_{\left(G\right)}$.

Step 5: Use the Formula (34) to calculate likelihood degrees $L({\tilde{w}}_{iH}^{\left(G\right)}\geq {\tilde{w}}_{jH}^{\left(G\right)})$ (i,j=1,2,...,n) and $L({\tilde{w}}_{iH}^{\left(p\right)}\geq {\tilde{w}}_{jH}^{\left(p\right)})$ (i,j=1,2,...,n,p=1,2,...,m).

Step 6: Establish likelihood degree matrices $L_{p}={\left(z_{ij}^{\left(p\right)}\right)}_{n\times n}$ (p=1,2,...,m) and $L_{G}={\left(z_{ij}^{\left(G\right)}\right)}_{n\times n}$ as per (35) and (36).

Step 7: Use (37) to determine the consensus index of the individual judgments in ${\tilde{A}}_{\left(p\right)}$ for each p=1,2,...,m. If $CD(L_{p})\geq c_{\delta }$ for all p=1,2,...,m, then go to the next step; otherwise, return the TFPR ${\tilde{A}}_{\left(q\right)}$ having $CD(L_{q})<c_{\delta }$ (q∈{1,2,...,m}) to the decision maker $d_{q}$ for a modification and go to step 10. 

Step 8: Sum values in the *i*th row of the group likelihood degree matrix $L_{G}$, and obtain ranking scores $s_{i}=\sum_{j=1}^{n}z_{ij}^{\left(G\right)}$ (i=1,2,...,n).

Step 9: Rank the alternatives in $X=\{x_{1},x_{2},...,x_{n}\}$ according to the decreasing order of the scores $s_{i}$ (i=1,2,...,n).

Step 10: End.

## 5. A Case Study of Selecting Energy-Saving and Low-Carbon Technology Schemes in Star Hotels

In this section, the proposed group decision making consensus model is applied to examine an energy-saving and low-carbon technology scheme selection problem for star hotels.

With the development of green tourism, numerous star hotels in China have faced the construction of a sustainable energy-saving and low-carbon system. An important stage in constructing such a system is to select the best one from multiple energy-saving and low-carbon technology schemes. According to the experts’ viewpoints and literature on energy-saving and low-carbon technologies, key criteria are identified and categorized into three groups as follows:

(1) $c_{1}$: Energy efficiency. Efficiencies of the considered energy equipment and the overall technical system are two important factors in selecting energy-saving and low-carbon technology schemes for star hotels. Energy efficiency has been widely acknowledged as a promising approach for tackling environmental issues, and thus improving energy efficiency in star hotels is becoming increasingly significant. Energy efficiency programs offer a development prospect of renewable energy requirements. The energy efficient equipment in star hotels includes energy saving light bulbs, boilers and cooling equipment with high efficiency, recovery systems, and so on.
(2) $c_{2}$: Capacity of energy-saving and carbon emission reduction. This capacity indicates the suitable performance of a technology scheme. The stronger the capacity, the better the technology scheme. Moreover, this criterion could be divided to two sub-criteria below.
  (i) $c_{21}$: Energy-saving capacity. This sub-criterion reflects the energy-saving performance and indicates how much energy is saved from the technology scheme.
  (ii) $c_{22}$: Low-carbon capacity. This sub-criterion reflects the low-carbon performance and shows how much carbon emission is reduced by the technology scheme.
(3) $c_{3}$: Economic effectiveness. To rank energy-saving and low-carbon technology schemes, the investment cost plays an important role. The main goal of this criterion is lower investment cost with better performance. Therefore, this criterion is often measured and reflected by investment payback periods of per unit energy-saving and per unit carbon emission reduction.

**Figure 1:**
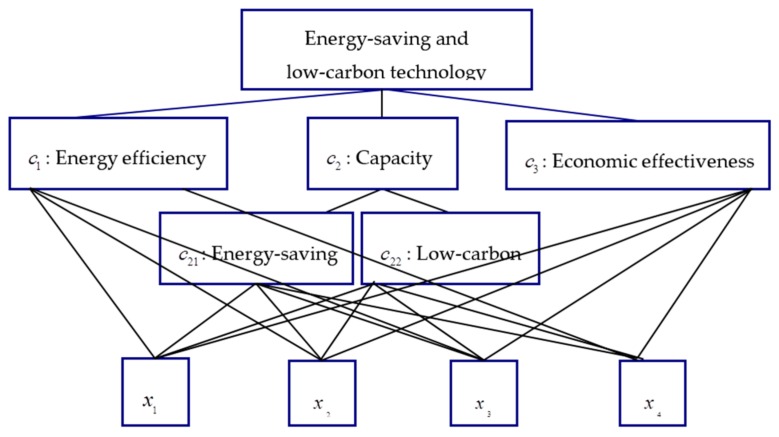
Decision Hierarchical structure.

After preliminary screening, four technology schemes $x_{i}$ (i=1,2,3,4) are determined by a star hotel as the evaluated alternatives. Hence, this MCDM problem can be structured as a hierarchy shown in Figure 1.

Assume that three experts $e_{1},e_{2}$, and $e_{3}$ are asked to evaluate the four technology schemes based on the above criteria and sub-criteria, and their importance weights are 04, 0.3 and 0.3, respectively. Each expert $e_{p}$ (p=1,2,3) employs the paired comparison method to elicit his/her fuzzy judgments for the four technology schemes with respect to each of the four criteria or sub-criteria $c_{1},c_{21},c_{22}$ and $c_{3}$, and structured these fuzzy judgments as TFPRs ${\tilde{A}}_{\left(p\right)}^{\Lambda }$ listed in Table (p+1), where $\Lambda \in \{c_{1},c_{21},c_{22},c_{3}\}$. 

According to the computation Formula (32), inconsistency indices of the individual TFPRs given in Table 2, Table 3 and Table 4 are determined and shown in the second column in Table 5.

Assume that an acceptable consistency threshold is set to be 0.3562, which is a geometric inconsistency approximated threshold value corresponding to 4×4 pairwise comparison matrices and CR=0.1 listed in Table 1. Thus, as per Definition 3, all individual TFPRs given in Table 2, Table 3 and Table 4 are of acceptable consistency. According to (33), group TFPRs are obtained and shown in Table 6.

By using the computation Formula (24), triangular fuzzy weights ${\tilde{w}}_{iH}$ (i=1,2,3,4) are determined and respectively listed in the last four columns in Table 5 for each individual TFPR. Similarly, group triangular fuzzy weights are derived from each group TFPR given in Table 6, and are shown in Table 7.

As per (34) and (35), twelve likelihood degree matrices are obtained from triangular fuzzy weights given in Table 5 for the individual TFPRs. By using (34) and (36), we can establish four likelihood degree matrices from the group triangular fuzzy weights shown in Table 7. According to (37), consensus indices of the individual judgments listed in Table 2, Table 3 and Table 4 are determined and shown in the third column in Table 5.

Assume that acceptable consensus thresholds are respectively set to be 0.8, 0.7, 0.65 and 0.75 for group decision making problems on the key criteria or sub-criteria $c_{1},c_{21},c_{22}$ and $c_{3}$. Then, it follows from the consensus indices given in Table 5 that each consensus index is more than or equal to its acceptable threshold. This implies that the three experts reach a consensus ranking order listed in the last column in Table 7.

In order to obtain a final ranking order of the four technology schemes, we need to aggregate triangular fuzzy weights in Table 7 into overall fuzzy weights of $x_{i}$ (i=1,2,3,4). Assume that importance weights of criteria or sub-criteria $c_{1},c_{21},c_{22}$ and $c_{3}$ are 0.35, 0.2, 0.15 and 0.3. Using triangular fuzzy geometric weighting method yields overall fuzzy weights as

$${\tilde{w}}_{x_{1}}=(0.492,0.578,0.705),\;{\tilde{w}}_{x_{2}}=(1.466,1.733,1.899)\;$$

$${\tilde{w}}_{x_{3}}=(1.108,1.302,1.498),\;{\tilde{w}}_{x_{4}}=(0.640,0.766,0.980)\;$$

According to (34), a likelihood degree matrix is established as

$$L_{O}={\left(L({\tilde{w}}_{x_{i}}\geq {\tilde{w}}_{x_{j}})\right)}_{4\times 4}=\left(\begin{array}{llll} 0.5 & 0 & 0 & 0\\ 1 & 0.5 & 1 & 1\\ 1 & 0 & 0.5 & 1\\ 1 & 0 & 0 & 0.5 \end{array}\right)\;$$

By summing values in the *i*th row of the above matrix $L_{O}$, we obtain ranking scores $s_{1}=0.5,s_{2}=3.5,s_{3}=2.5$ and $s_{4}=1.5$. As $s_{2}>s_{3}>s_{4}>s_{1}$, the four technology schemes are ranked as $x_{2}\overset{100\%}{\underline{≻}}x_{3}\overset{100\%}{\underline{≻}}x_{4}\overset{100\%}{\underline{≻}}x_{1}$, and thus $x_{2}$ is the best technology scheme.

## 6. Conclusions

This paper has developed a triangular fuzzy group consensus decision making model. This model takes both acceptable consistency of individual judgments and acceptable consensus of individual and group decision results into consideration. A consistency index has been proposed to measure inconsistency of TFPRs. A possibility degree-based index has been devised to measure consensus of individual and group ranking orders of all alternatives. A case study of selecting energy-saving and low-carbon technology schemes in star hotels has been offered to examine the application of the group consensus decision making model developed.

Some significant issues could be addressed in the future. For instance, sometimes, a decision maker may provide extremely fuzzy judgments in a TFPR while this TFPR may be judged to be acceptable under the proposed acceptable consistency model. In addition, some judgments in an original TFPR may be missing. It is worth examining how the simplified computation formulas and the proposed consistency index are adapted and extended to handle these cases.

## Figures and Tables

**Table 1:** Geometric inconsistency approximated thresholds.

Consistency Ratio (CR)	0.01	0.05	0.1	0.15
Threshold value (*n* = 3)	0.0314	0.1573	0.3147	0.4720
Threshold value (*n* = 4)	0.0352	0.1763	0.3562	0.5289
Threshold value (*n* > 4)	~0.037	~0.185	~0.370	~0.555

**Table 2:** Triangular fuzzy judgments provided by the expert $e_{1}$.

TFPR		$x_{1}\;$	$x_{2}\;$	$x_{3}\;$	$x_{4}\;$
${\tilde{A}}_{\left(1\right)}^{c_{1}}$	$x_{1}$	1	(1/4, 1/3, 1/2)	(1/4, 1/3, 1/2)	(1/3, 1/2, 1)
	$x_{2}$	(2, 3, 4)	1	(3/2, 3/2, 2)	(1, 2, 3)
	$x_{3}$	(2, 3, 4)	(1/2, 2/3, 2/3)	1	(1, 2, 3)
	$x_{4}$	(1, 2, 3)	(1/3, 1/2, 1)	(1/3, 1/2, 1)	1
${\tilde{A}}_{\left(1\right)}^{c_{21}}$	$x_{1}$	1	(2/3, 5/6, 1)	(4/7, 5/7, 6/7)	(1, 3/2, 2)
	$x_{2}$	(1, 6/5, 3/2)	1	(4/7, 6/7, 1)	(1, 2, 5/2)
	$x_{3}$	(7/6, 7/5, 7/4)	(1, 7/6, 7/4)	1	(3/2, 7/4, 5/2)
	$x_{4}$	(1/2, 2/3, 1)	(2/5, 1/2, 1)	(2/5, 4/7, 2/3)	1
${\tilde{A}}_{\left(1\right)}^{c_{22}}$	$x_{1}$	1	(1/3, 1/2, 1)	(1/4, 1/3, 1/2)	(5/4, 2, 3)
	$x_{2}$	(1, 2, 3)	1	(1/2, 2/3, 1)	(3, 4, 5)
	$x_{3}$	(2, 3, 4)	(1, 3/2, 2)	1	(5, 6, 7)
	$x_{4}$	(1/3, 1/2, 4/5)	(1/5, 1/4, 1/3)	(1/7, 1/6, 1/5)	1
${\tilde{A}}_{\left(1\right)}^{c_{3}}$	$x_{1}$	1	(1/5, 1/4, 1/3)	(1/5, 1/4, 1/3)	(1/6, 1/5, 1/4)
	$x_{2}$	(3, 4, 5)	1	(3/2, 3/2, 2)	(1, 2, 3)
	$x_{3}$	(3, 4, 5)	(1/2, 2/3, 2/3)	1	(1, 2, 3)
	$x_{4}$	(4, 5, 6)	(1/3, 1/2, 1)	(1/3, 1/2, 1)	1

**Table 3:** Triangular fuzzy judgments provided by the expert $e_{2}$.

TFPR		$x_{1}\;$	$x_{2}\;$	$x_{3}\;$	$x_{4}\;$
${\tilde{A}}_{\left(2\right)}^{c_{1}}$	$x_{1}$	1	(1/3, 1/2, 1)	(1/3, 2/3, 1)	(1, 4/3, 3/2)
	$x_{2}$	(1, 2, 3)	1	(1, 3/2, 2)	(2, 3, 7/2)
	$x_{3}$	(1, 3/2, 3)	(1/2, 2/3, 1)	1	(1, 2, 3)
	$x_{4}$	(2/3, 3/4, 1)	(2/7, 1/3, 1/2)	(1/3, 1/2, 1)	1
${\tilde{A}}_{\left(2\right)}^{c_{21}}$	$x_{1}$	1	(1/4, 2/7, 3/7)	(1/5, 2/5, 3/5)	(1/9, 2/9, 1/3)
	$x_{2}$	(7/3, 7/2, 4)	1	(6/5, 7/5, 8/5)	(2/3, 7/9, 8/9)
	$x_{3}$	(5/3, 5/2, 5)	(5/8, 5/7, 5/6)	1	(4/9, 2/3, 1)
	$x_{4}$	(3, 9/2, 9)	(9/8, 9/7, 3/2)	(1, 3/2, 9/4)	1
${\tilde{A}}_{\left(2\right)}^{c_{22}}$	$x_{1}$	1	(5/3, 2, 3)	(1/2, 5/8, 2/3)	(1, 5/4, 5/3)
	$x_{2}$	(1/3, 1/2, 3/5)	1	(2/7, 1/3, 1/2)	(1/2, 3/4, 1)
	$x_{3}$	(3/2, 8/5, 2)	(2, 3, 7/2)	1	(1, 2, 3)
	$x_{4}$	(3/5, 4/5, 1)	(1, 4/3, 2)	(1/3, 1/2, 1)	1
${\tilde{A}}_{\left(2\right)}^{c_{3}}$	$x_{1}$	1	(1/5, 2/7, 3/7)	(3/2, 2, 3)	(1/4, 1/3, 1/2)
	$x_{2}$	(7/3, 7/2, 5)	1	(6, 7, 8)	(1, 3/2, 2)
	$x_{3}$	(1/3, 1/2, 2/3)	(1/8, 1/7, 1/6)	1	(1/7, 1/6, 1/5)
	$x_{4}$	(2, 3, 4)	(1/2, 2/3, 1)	(5, 6, 7)	1

**Table 4:** Triangular fuzzy judgments provided by the expert $e_{3}$.

TFPR		$x_{1}\;$	$x_{2}\;$	$x_{3}\;$	$x_{4}\;$
${\tilde{A}}_{\left(3\right)}^{c_{1}}$	$x_{1}$	1	(1/7, 1/6, 1/5)	(1/6, 1/5, 1/4)	(1/2, 1, 5/4)
	$x_{2}$	(5, 6, 7)	1	(1/2, 1, 6/5)	(5, 6, 7)
	$x_{3}$	(4, 5, 6)	(5/6, 1, 2)	1	(4, 5, 6)
	$x_{4}$	(4/5, 1, 2)	(1/7, 1/6, 1/5)	(1/6, 1/5, 1/4)	1
${\tilde{A}}_{\left(3\right)}^{c_{21}}$	$x_{1}$	1	(1/4, 2/7, 3/7)	(1/4, 1/3, 1/2)	(1, 6/5, 3/2)
	$x_{2}$	(7/3, 7/2, 4)	1	(1, 7/6, 3/2)	(3, 7/2, 4)
	$x_{3}$	(2, 3, 4)	(2/3, 6/7, 1)	1	(2, 3, 4)
	$x_{4}$	(2/3, 5/6, 1)	(1/4, 2/7, 1)	(1/4, 1/3, 1/2)	1
${\tilde{A}}_{\left(3\right)}^{c_{22}}$	$x_{1}$	1	(1/4, 1/3, 1/2)	(1/3, 3/7, 1/2)	(2/3, 2/3, 4/3)
	$x_{2}$	(2, 3, 4)	1	(1, 8/7, 4/3)	(2, 8/3, 4)
	$x_{3}$	(2, 7/3, 3)	(3/4, 7/8, 1)	1	(2, 7/3, 7/2)
	$x_{4}$	(3/4, 3/2, 3/2)	(1/4, 3/8, 1/2)	(2/7, 3/7, 1/2)	1
${\tilde{A}}_{\left(3\right)}^{c_{3}}$	$x_{1}$	1	(2/7, 1/3, 3/5)	(1/3, 1/2, 4/5)	(2/3, 1, 3/2)
	$x_{2}$	(5/3, 3, 7/2)	1	(3/2, 2, 3)	(2, 3, 4)
	$x_{3}$	(5/4, 2, 3)	(1/3, 1/2, 2/3)	1	(3/2, 2, 3)
	$x_{4}$	(2/3, 1, 3/2)	(1/4, 1/3, 1/2)	(1/3, 1/2, 2/3)	1

**Table 5:** Inconsistency and consensus indices as well as triangular fuzzy weights.

TFPR	Inconsistency Index	Consensus Index	${\tilde{w}}_{1H}\;$	${\tilde{w}}_{2H}\;$	${\tilde{w}}_{3H}\;$	${\tilde{w}}_{4H}\;$
${\tilde{A}}_{\left(1\right)}^{c_{1}}$	0.0507	0.9758	(0.421, 0.485, 0.638)	(1.536, 1.730, 1.897)	(1.167, 1.410, 1.441)	(0.579, 0.841, 1.313)
${\tilde{A}}_{\left(1\right)}^{c_{21}}$	0.0079	0.7028	(0.871, 0.972, 1.032)	(0.919, 1.198, 1.316)	(1.279, 1.300, 1.496)	(0.546, 0.661, 0.881)
${\tilde{A}}_{\left(1\right)}^{c_{22}}$	0.0049	1.000	(0.578, 0.760, 1.088)	(1.178, 1.520, 1.848)	(2.036, 2.280, 2.389)	(0.358, 0.380, 0.420)
${\tilde{A}}_{\left(1\right)}^{c_{3}}$	0.1732	0.9167	(0.331, 0.334, 0.352)	(1.592, 1.860, 2.141)	(1.210, 1.520, 1.627)	(0.817, 1.057, 1.564)
${\tilde{A}}_{\left(2\right)}^{c_{1}}$	0.0295	0.8576	(0.630, 0.816, 1.014)	(1.339, 1.730, 1.901)	(0.885, 1.189, 1.645)	(0.586, 0.595, 0.720)
${\tilde{A}}_{\left(2\right)}^{c_{21}}$	0.0249	0.7972	(0.273, 0.399, 0.540)	(1.259, 1.397, 1.434)	(0.884, 1.045, 1.334)	(1.452, 1.716, 2.191)
${\tilde{A}}_{\left(2\right)}^{c_{22}}$	0.0239	0.6667	(1.097, 1.118, 1.177)	(0.507, 0.595, 0.682)	(1.409, 1.760, 1.999)	(0.685, 0.855, 1.161)
${\tilde{A}}_{\left(2\right)}^{c_{3}}$	0.0094	0.7500	(0.534, 0.661, 0.877)	(2.077, 2.462, 2.786)	(0.314, 0.330, 0.341)	(1.609, 1.860, 2.137)
${\tilde{A}}_{\left(3\right)}^{c_{1}}$	0.0503	0.9103	(0.353, 0.427, 0.468)	(2.036, 2.450, 2.558)	(2.033, 2.240, 2.737)	(0.397, 0.427, 0.527)
${\tilde{A}}_{\left(3\right)}^{c_{21}}$	0.0778	0.8641	(0.549, 0.567, 0.686)	(1.878, 1.897, 1.917)	(1.376, 1.626, 1.858)	(0.446, 0.518, 0.852)
${\tilde{A}}_{\left(3\right)}^{c_{22}}$	0.0098	0.6968	(0.512, 0.579, 0.720)	(1.514, 1.811, 2.007)	(1.485, 1.539, 1.595)	(0.498, 0.730, 0.756)
${\tilde{A}}_{\left(3\right)}^{c_{3}}$	0.0343	0.8204	(0.540, 0.639, 0.857)	(1.669, 2.060, 2.281)	(0.976, 1.190, 1.426)	(0.537, 0.639, 0.760)

**Table 6:** The obtained group triangular fuzzy judgments.

TFPR		$x_{1}\;$	$x_{2}\;$	$x_{3}\;$	$x_{4}\;$
${\tilde{A}}_{\left(G\right)}^{c_{1}}$	$x_{1}$	1	(0.230, 0.306, 0.468)	(0.241, 0.352, 0.500)	(0.523, 0.826, 1.208)
	$x_{2}$	(2.137, 3.268, 4.348)	1	(0.955, 1.328, 1.716)	(1.995, 3.140, 4.051)
	$x_{3}$	(2.000, 2.841, 4.419)	(0.583, 0.753, 1.047)	1	(1.516, 2.633, 3.693)
	$x_{4}$	(0.828, 1.211, 1.912)	(0.247, 0.318, 0.501)	(0.271, 0.380, 0.660)	1
${\tilde{A}}_{\left(G\right)}^{c_{21}}$	$x_{1}$	1	(0.370, 0.438, 0.601)	(0.325, 0.478, 0.655)	(0.517, 0.791, 1.072)
	$x_{2}$	(1.664, 2.283, 2.703)	1	(0.844, 1.089, 1.300)	(1.231, 1.782, 2.111)
	$x_{3}$	(1.527, 2.092, 3.077)	(0.769, 0.918, 1.185)	1	(1.135, 1.540, 2.187)
	$x_{4}$	(0.933, 1.264, 1.934)	(0.474, 0.561, 0.812)	(0.457, 0.649, 0.881)	1
${\tilde{A}}_{\left(G\right)}^{c_{22}}$	$x_{1}$	1	(0.496, 0.671, 1.130)	(0.336, 0.434, 0.545)	(0.968, 1.250, 1.972)
	$x_{2}$	(0.885, 1.490, 2.018)	1	(0.521, 0.637, 0.886)	(1.553, 2.141, 2.882)
	$x_{3}$	(1.835, 2.304, 2.980)	(1.129, 1.571, 1.921)	1	(2.342, 3.247, 4.405)
	$x_{4}$	(0.507, 0.800, 1.033)	(0.347, 0.467, 0.644)	(0.227, 0.308, 0.427)	1
${\tilde{A}}_{\left(G\right)}^{c_{3}}$	$x_{1}$	1	(0.223, 0.284, 0.429)	(0.427, 0.574, 0.838)	(0.285, 0.378, 0.527)
	$x_{2}$	(2.331, 3.521, 4.484)	1	(2.274, 2.596, 3.424)	(1.231, 2.072, 2.896)
	$x_{3}$	(1.193, 1.742, 2.342)	(0.292, 0.385, 0.440)	1	(0.630, 0.949, 1.331)
	$x_{4}$	(1.898, 2.646, 3.509)	(0.345, 0.483, 0.812)	(0.751, 1.054, 1.587)	1

**Table 7:** Triangular fuzzy weights of group TFPRs.

TFPR	${\tilde{w}}_{1H}^{\left(G\right)}\;$	${\tilde{w}}_{2H}^{\left(G\right)}\;$	${\tilde{w}}_{3H}^{\left(G\right)}\;$	${\tilde{w}}_{4H}^{\left(G\right)}\;$	Ranking
${\tilde{A}}_{\left(G\right)}^{c_{1}}$	(0.451, 0.546, 0.667)	(1.608, 1.921, 2.072)	(1.263, 1.541, 1.857)	(0.520, 0.618, 0.831)	$x_{2}\overset{100\%}{\underline{≻}}x_{3}\overset{100\%}{\underline{≻}}x_{4}\overset{85.5\%}{\underline{≻}}x_{1}$
${\tilde{A}}_{\left(G\right)}^{c_{21}}$	(0.528, 0.638, 0.762)	(1.284, 1.451, 1.474)	(1.154, 1.311, 1.564)	(0.708, 0.824, 1.027)	$x_{2}\overset{78.3\%}{\underline{≻}}x_{3}\overset{100\%}{\underline{≻}}x_{4}\overset{100\%}{\underline{≻}}x_{1}$
${\tilde{A}}_{\left(G\right)}^{c_{22}}$	(0.676, 0.777, 0.984)	(0.990, 1.194, 1.405)	(1.658, 1.852, 2.006)	(0.480, 0.582, 0.680)	$x_{3}\overset{100\%}{\underline{≻}}x_{2}\overset{100\%}{\underline{≻}}x_{1}\overset{100\%}{\underline{≻}}x_{4}$
${\tilde{A}}_{\left(G\right)}^{c_{3}}$	(0.443, 0.498, 0.605)	(1.749, 2.086, 2.360)	(0.757, 0.893, 0.979)	(0.882, 1.077, 1.384)	$x_{2}\overset{100\%}{\underline{≻}}x_{4}\overset{100\%}{\underline{≻}}x_{3}\overset{100\%}{\underline{≻}}x_{1}$
